# Remarkable response to alectinib for metastatic papillary thyroid cancer with STRN-ALK fusion: A case report

**DOI:** 10.3389/fonc.2022.1009076

**Published:** 2022-11-09

**Authors:** Lucheng Zhu, Shenglin Ma, Bing Xia

**Affiliations:** ^1^ Department of Thoracic Oncology, Affiliated Hangzhou Cancer Hospital, Zhejiang University School of Medicine, Hangzhou, China; ^2^ Department of Oncology, Affiliated Hangzhou Cancer Hospital, Zhejiang Chinese Medical University, Hangzhou, China; ^3^ Cancer Center, Zhejiang University, Hangzhou, China

**Keywords:** ALK inhibitor, STRN-ALK fusion, thyroid cancer, alectinib, case report

## Abstract

Little is known about the efficacy of alectinib for papillary thyroid cancer with STRN-ALK fusion. A 64-year-old female presented with metastatic papillary thyroid cancer, widespread to lungs, mediastinal lymph nodes and brain 20 years after surgery. Disease progression still occurred after radioactive iodine therapy, chemotherapy, and radiotherapy. Tissue obtained from left cervical lymph node confirmed metastatic papillary thyroid cancer. Molecular profiling from re-biopsy tissue identified an *STRN-ALK* fusion rearrangement. After multidisciplinary discussion, alectinib was administered to the patient. Treatment was well tolerated, and follow-up images confirmed a partial response. ALK occurs rarely, with limited data suggesting the efficacy of ALK inhibitors in thyroid cancer. We presented the first case of a patient with PTC and STRN-ALK fusion to be treat effectively with alectinib.

## Introduction

Anaplastic lymphoma kinase (ALK) rearrangement can be detected in various solid tumors, including non-small cell lung cancer, colon cancer, kidney cancer (1). ALK rearrangement is seen in about 5% of all non-small cell lung cancer patients (2). In thyroid cancer, data from TCGA showed the prevalence of ALK rearrangement was 0.8% (3). Currently, little is known about the efficacy of ALK inhibitors on patients with STRN-ALK fusion tumors. Here we present a case showing remarkable response to alectinib for metastatic pulmonary metastases from papillary thyroid cancer with STRN-ALK fusion.

## Case presentation

**Figure 1:**
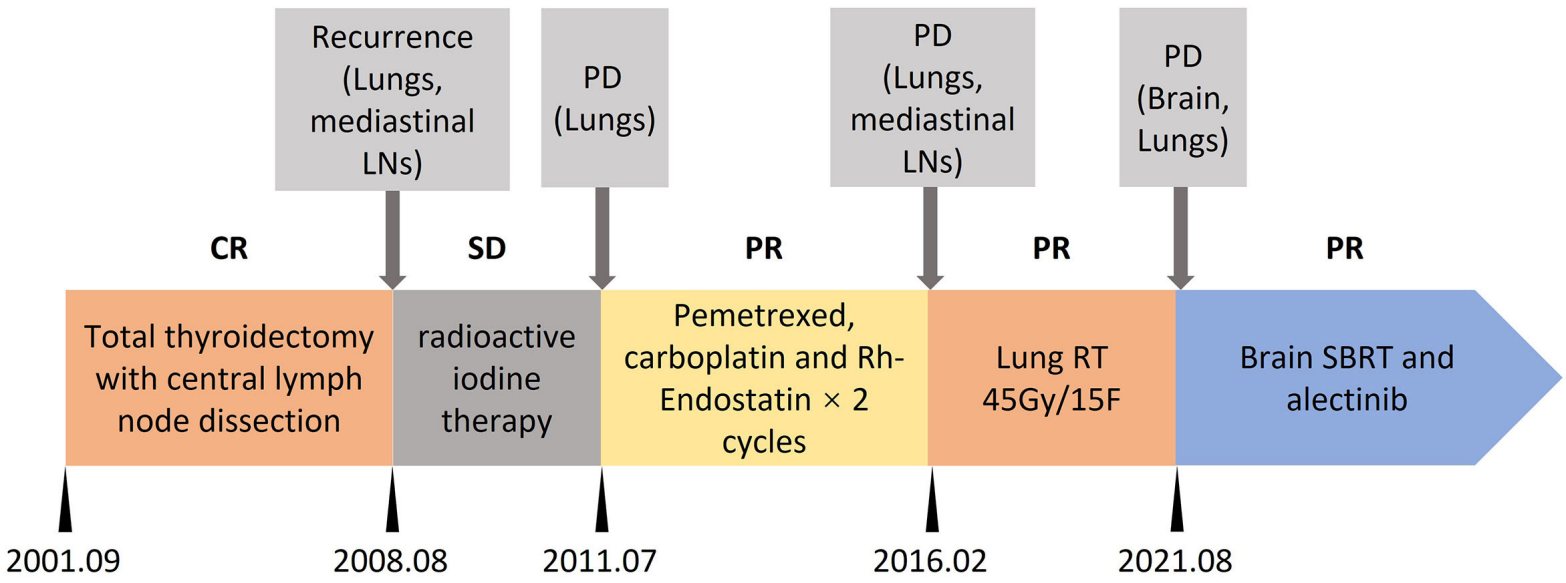
Treatment timeline. CR, complete response; SD, stable disease; PD, progression disease; PR, partial response; LNs, lymph nodes; RT, radiotherapy; SBRT, stereotactic body radiotherapy.

A 64-year-old female was affected by a metastatic papillary thyroid cancer (PTC), widespread to lungs, mediastinal lymph nodes and brain. The treatment timeline is shown in **Figure 1**. Over the 20 years from the initial diagnosis, the patient underwent total thyroidectomy with central lymph node dissection. The patient also received 2 cycles of radioactive iodine therapy after surgery. After 7 years from surgery, the patient experienced progression of disease (lungs and mediastinal lymph nodes); therefore, another cycle of radioactive iodine therapy was performed. Three years after, the patient experienced progression of disease again (lungs) in 2011. Tissue obtained from lung lesions confirmed metastatic thyroid cancer. Two cycles of pemetrexed (500mg/m2, day 1), carboplatin (AUC=5, day1) and Rh-Endostatin (15mg days 1 to 14) were administrated. The patient showed a partial response of the disease, without grade 3 to 4 adverse events. After 5 years of treatment, progression was confirmed by computed tomography scan (lungs). She also experienced cough and hemoptysis. Imaging showed lung mass near the bronchus, responsible for hemoptysis. Thoracic radiotherapy was carried out with stabilization of the lung metastases and absence of hemoptysis symptoms. Disease remained stable for about 5 years until brain metastasis appeared.

Brain magnetic resonance imaging revealed brain metastasis (right frontal lobe). The patient experienced headache. Fractionate stereotactic radiotherapy (FSRT) was performed with brain metastasis. Headache was relief after FSRT. A computed tomography scan revealed progression of disease in lungs and cervical lymph nodes. Tissue obtained from left cervical lymph node confirmed metastatic papillary thyroid cancer with the immunohistochemistry results of CK (+), TTF-1 (+), CEA (-), and Tg (+). The DNA of re-biopsy tissue was profiled with a capture-based targeted DNA sequencing panel (Burning Rock Biotech, Guangzhou, China) that targets 18 genes (BRAF, NRAS, HRAS, KRAS, RET, NTRK1, ETV6, ALK, PPARG, TERT, EIF1AX, PTEN, AKT1, PIK3CA, TP53, CTNNB1, TSHR, and GNAS) and spans 140 kb of the human genome. *STRN-ALK* fusion rearrangement (S3:A20) with variant allele fraction (VAF) 33.72% and NRAS (1p13.2) copy number loss were identified by targeted NGS of the biopsy sample.

**Figure 2:**
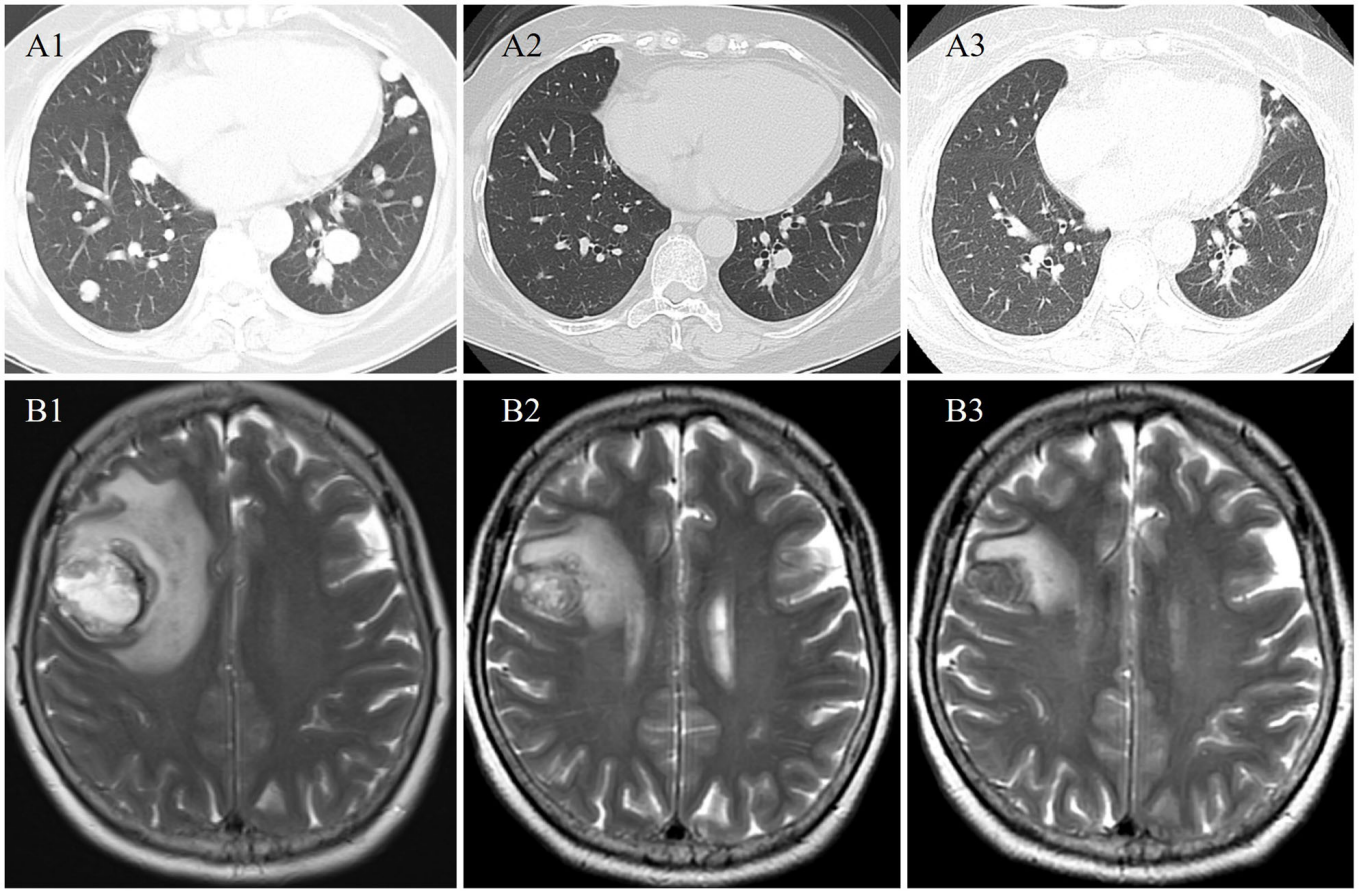
Radiological evaluation at baseline (A1 and B1), 1 (A2 and B2), and 3 (A3 and B3) months from start of alectinib therapy demonstrating an ongoing partial response in the pulmonary and brain metastases.

After multidisciplinary discussion and consent to treatment by the patient, alectinib (600mg twice daily) was administered to the patient. CT and MR scans showed radiological partial response at 1 and 3 months on treatment (**Figure 2**). The treatment was well tolerated, except for grade 1 diarrhea. To date, the duration of response is more than 8 months. She continues on alectinib.

## Discussion

PROFILE 1014 showed ALK inhibitor crizotinib prolonged the survival time compared to chemotherapy in ALK rearrangement NSCLC patients (4). ALEX study showed alectinib had a significantly longer PFS than crizotinib (5). Moreover, the OS benefit of alectinib was seen in patients with brain metastases. STRN-ALK was found as the most common type of ALK fusion (6). Kelly et al. showed ALK inhibitors suppressed thyroid cancer cells harboring STRN-ALK fusion proliferation (6). Currently, there is no randomized control study targeting ALK rearrangement in thyroid cancer. A few researchers found dramatic response of ALK inhibitors in ALK rearrangement thyroid patients. Aydemirli et al. reported a partial response in an EML-ALK fusion patient treated with lorlatinib after resistance to crizotinib (7). de Salins et al. reported a complete response in an ALK rearrangement patient treated with crizotinib (8). In lung cancer, breast cancer and peritoneal mesothelioma, reports had showed the dramatic response of alectinib in patients with STRN-ALK fusion (9–11). While the value of alectinib in patients with STRN-ALK fusion thyroid cancer was unknown.

Currently, more than 90 distinct ALK fusion types have been identified in ALK+ tumors (12). Their response to ALK inhibitors varied differently. Furugaki et al. evaluated the sensitivity to alectinib of eight ALK+ type (EML4-ALK variant 1-4, KIF5B-ALK, KLC1-ALK, STRN-ALK, and TFG-ALK) and no difference were found between them (13).

## Conclusion

ALK occurs rarely, with limited data suggesting the efficacy of ALK inhibitors in thyroid cancer. To the best of our knowledge, we presented the first case of a patient with PTC and STRN-ALK fusion to be treat effectively with alectinib.

## Data availability statement

The original contributions presented in the study are included in the article/supplementary material. Further inquiries can be directed to the corresponding authors.

## Ethics statement

The studies involving human participants were reviewed and approved by The Scientific Research Board of Hangzhou Cancer Hospital. The patients/participants provided their written informed consent to participate in this study.

## Author contributions

LZ, BX, SM: Conceptualization, Writing- Original draft preparation; LZ: Investigation; BX: Project administration; LZ, BX, SM: Writing- Reviewing and Editing. All authors contributed to the article and approved the submitted version.

## Funding

This study was supported by grants from Key Project of Hangzhou. The funder had no role in study design, data collection and analysis, decision to publish, or preparation of the manuscript.

## Acknowledgments

The authors thank the patient for her participation in this study.

## Conflict of interest

The authors declare that the research was conducted in the absence of any commercial or financial relationships that could be construed as a potential conflict of interest.

## Publisher’s note

All claims expressed in this article are solely those of the authors and do not necessarily represent those of their affiliated organizations, or those of the publisher, the editors and the reviewers. Any product that may be evaluated in this article, or claim that may be made by its manufacturer, is not guaranteed or endorsed by the publisher.

## References

[1] Kruczynski A Delsol G Laurent C Brousset P Lamant L. Anaplastic lymphoma kinase as a therapeutic target. Expert Opin Ther Targets (2012) 16(11):1127–38. doi: 10.1517/14728222.2012.719498 22998583

[2] Du X Shao Y Qin HF Tai YH Gao HJ. ALK-rearrangement in non-small-cell lung cancer (NSCLC). Thorac Cancer (2018) 9(4):423–30. doi: 10.1111/1759-7714.12613 PMC5879058 29488330

[3] Cancer Genome Atlas Research Network. Integrated genomic characterization of papillary thyroid carcinoma. Cell (2014) 159(3):676–90. doi: 10.1016/j.cell.2014.09.050 PMC4243044 25417114

[4] Solomon BJ Kim DW Wu YL Nakagawa K Mekhail T Felip E. Final overall survival analysis from a study comparing first-line crizotinib versus chemotherapy in ALK-Mutation-Positive non-Small-Cell lung cancer. J Clin Oncol (2018) 36(22):2251–8. doi: 10.1200/JCO.2017.77.4794 29768118

[5] Mok T Camidge DR Gadgeel SM Rosell R Dziadziuszko R Kim DW. Updated overall survival and final progression-free survival data for patients with treatment-naive advanced ALK-positive non-small-cell lung cancer in the ALEX study. Ann Oncol (2020) 31(8):1056–64. doi: 10.1016/j.annonc.2020.04.478 32418886

[6] Kelly LM Barila G Liu P Evdokimova VN Trivedi S Panebianco F. Identification of the transforming STRN-ALK fusion as a potential therapeutic target in the aggressive forms of thyroid cancer. Proc Natl Acad Sci USA (2014) 111(11):4233–8. doi: 10.1073/pnas.1321937111 PMC3964116 24613930

[7] Aydemirli MD van Eendenburg JDH van Wezel T Oosting J Corver WE Kapiteijn E. Targeting EML4-ALK gene fusion variant 3 in thyroid cancer. Endocr Relat Cancer (2021) 28(6):377–89. doi: 10.1530/ERC-20-0436 PMC8183637 33878728

[8] de Salins V Loganadane G Joly C Abulizi M Nourieh M Boussion H. Complete response in anaplastic lymphoma kinase-rearranged oncocytic thyroid cancer: A case report and review of literature. World J Clin Oncol (2020) 11(7):495–503. doi: 10.5306/wjco.v11.i7.495 32821654 PMC7407927

[9] Nagasaka M Sarvadevabatla N Iwata S Ge Y Sukari A Klosowski C. STRN-ALK, a novel in-frame fusion with response to alectinib. JTO Clin Res Rep (2021) 2(2):100125. doi: 10.1016/j.jtocrr.2020.100125 34589985 PMC8474477

[10] Kelly AD Wiklund T Kononen J Creeden J. STRN-ALK fusion-positive case of breast cancer with response to alectinib. JCO Precis Oncol (2021) 5 :1281-84. doi: 10.1200/PO.21.00142 PMC8373546 34423228

[11] Rüschoff JH Gradhand E Kahraman A Rees H Ferguson JL Curioni-Fontecedro A. STRN -ALK rearranged malignant peritoneal mesothelioma with dramatic response following ceritinib treatment. JCO Precis Oncol (2019) 3:1–6. doi: 10.1200/PO.19.00048 PMC7446511 32914035

[12] Ou SI Zhu VW Nagasaka M. Catalog of 5' fusion partners in ALK-positive NSCLC circa 2020. JTO Clin Res Rep (2020) 1(1):100015. doi: 10.1016/j.jtocrr.2020.100015 34589917 PMC8474466

[13] Furugaki K Harada N Yoshimura Y. Sensitivity of eight types of ALK fusion variant to alectinib in ALK-transformed cells. Anticancer Drugs (2022) 33(2):124–31. doi: 10.1097/CAD.0000000000001249 PMC8734629 34520436

